# Updating the EU Directives on Midwifery: A generational opportunity for the profession and women’s rights

**DOI:** 10.18332/ejm/220333

**Published:** 2026-04-30

**Authors:** Daniela Drandic, Anna af Ugglas, Jacqueline Dunkley-Bent, Lia Brigante, Mervi Jokinen, Viktoria Vivilaki

**Affiliations:** 1 International Confederation of Midwives (ICM), The Hague, The Netherlands; 2 European Forum of National Nursing and Midwifery Associations, Copenhagen, Denmark; 3 European Midwives Association, Antwerp, Belgium

**Keywords:** midwife, midwifery, women's health, EU Directive on Minimum Professional Qualifications, SRMNAH

The European Union (EU) and European Economic Area (EEA) is a group of 30 countries, including the EU and three additional countries. Known as EU+EEA, these countries align with EU Directives across different policy areas. Midwives are one of seven regulated professions included in the Directive on Professional Qualifications (Directive 2005/36/EC), which sets the rules on EU-level minimum harmonized education requirements for these professions^1^. These rules exist to ensure free movement of the labor workforce across the EU+EEA countries, by ensuring that countries can easily recognize qualifications from other EU+EEA countries, knowing that educational programs all satisfy an agreed-upon minimum standard^2^. While individual countries in the EU can set higher standards for their midwifery education programs, they must not go below the minimum threshold. For this reason, the EU Directive on Minimum Professional Qualifications for Midwives (hereinafter referred to as the Directive) is critical to the development of the midwifery profession and quality of midwifery care in Europe.

A process to update the Directive began in 2024. This is a technical process whereby two sections of the Directive that describe the education of midwives [Article 40(3) The Training of Midwives and Annex V, point 5.5.1 Training Programme for Midwives], and consequently their scope of practice, are eligible for update. Other sections that define the minimum length of midwifery education and access to education programs, specifically in Article 40(1) and 40(2), are not eligible for update in the current process.

## The Directive

First written in 1981, the sections currently under review in the Directive have not been significantly updated since first written over 40 years ago^3^, despite developments in the profession, scientific research and professional activities that have happened since. In its current form, the Directive does not reflect the technological and scientific achievements that the profession has achieved in the last four decades, nor does it reflect the wealth of evidence supporting the implementation and enabling of robust midwifery services across sexual, reproductive, maternal, newborn and adolescent health services. The current Directive text lacks clarity and, as a result, is difficult to translate across the EU+EEA languages, and from policy into practice, hindering the full potential of midwives to contribute to the health of women in the EU and contribute to EU strategies and documents relating to sexual and reproductive health^4,5^ and gender equality^6^. Furthermore, it lacks alignment with international standards like the ICM Essential Competencies for Midwifery Practice^7^ and the ICM Global Standards for Education^8^, as well as recent WHO guidelines^9^, the recognized global benchmarks.

## The process

The EU Commission has delegated powers to change certain parts of the EU Directive, as described above. This is considered a technical, not legislative process, and as a result the EU Parliament is not involved.

In 2024, a tender to prepare a report on whether the EU Directive should be updated^10^ was awarded to an external consulting firm based in Brussels. The tender described the framework for the methodology the consulting firm should use to conduct the update. The tenderer nominated national experts from EU and EEA countries to coordinate the process, and mapped stakeholders including ministries of health, professional associations, educational institutions, regulatory and registration bodies for midwifery across the 30 countries of the EU/EEA. These stakeholders were given questionnaires on scientific and technological advancements in the field of midwifery, which they were asked to complete and return. Depending on their role in midwifery, they were asked to describe retrospectively how these advancements had been implemented in their teaching curricula, regulation or policy, as opposed to being asked to describe and provide evidence for updates to the Directive itself. An additional flaw in the methodology was that the surveys were not formally validated or culturally adapted for the diverse contexts of midwifery practice and education across the EU/EEA, potentially impacting the reliability and representativeness of the collected data. Finally, the methodology was also built around the premise that the final report would only include suggested changes to the Directive if 18 or more countries in the EU+EEA reported a topic in their work retrospectively^11^, as opposed to taking on scientific evidence on advancements.

The draft report was shared to all stakeholders by the tenderer, and selected stakeholders were invited to an in-person workshop in Brussels to provide direct feedback on the draft, which took place in October 2024. The criteria for the choice of stakeholders at the in-person workshop are not known, nor was it clear whose feedback would be included in the final report, and according to which criteria.

## Issues with the draft report

At the stakeholder meeting in Brussels on 22 October 2024, many concerns were voiced about problems with the methodology used to prepare the draft report. These included:

- Those who participated in the consultation were not informed of the purpose and methodology of the survey, and, as a result, there were gaps in the information provided;
- There were many responses that were misinterpreted or thematically misplaced, e.g. physiology and psychology were often misunderstood and placed under the ‘mental health’ theme, when in fact, they are very different; and
- The questionnaires were only available in English, and many stakeholders could not meaningfully participate as a result.

One of the main issues in the methodology was that the premise for changes to be included in the draft update of the Directive was based on 18 countries reporting that their midwifery education and regulation standards are above the minimum the EU Directive sets. As organizations who bring together professional associations of midwives and as individual experts in midwifery in Europe, we are aware that in at least half of the EU/EEA countries, the Directive is interpreted as the maximum, rather than the minimum standard for the training of midwives. This rigidity in methodology, which does not take into account the lived reality in EU/EEA countries, puts the midwifery profession in a very difficult position; on the one hand, more than half of countries interpret the Directive as their maximum standard, while on the other, updates to the Directive depend on the opposite – more than half the countries going above and beyond the standard. This hinders the development of the profession based on scientific evidence and advancements, hinders its autonomy, but also limits the mobility of the midwifery workforce across EU/EEA countries. Finally, the surveys and participation in the process were only available in English, which hinders meaningful involvement of all countries.

## The final report and next steps

The tenderer’s final report to the Commission was published in July 2025, and reflected only small, cosmetic changes to the Directive, which do not align it to scientific and technological advances in the field^12^. While the initial hope was that the Commission would make its final decision on updates to the Directive 2026, it has not been included in this year’s Commission Agenda^13^.

There have also been problems at the organizational level, specifically that the Directive update was initially being conducted by the Directorate General for Internal Market, Industry, Entrepreneurship and Small and Middle-sized Enterprises (DG-GROW) of the European Commission^10^, but was later moved to the portfolio of Employment, Social Affairs and Inclusion (DG-EMPL), further exacerbating advocacy efforts.

At the time of writing this editorial, there is no clear pathway for stakeholders like professional associations to engage with the Commission to ensure that the final text of the Directive reflects actual scientific and technological advances, ensuring midwifery in the EU+EEA maintains its competitiveness and global relevance.

## Collaboration between professional organizations

Motivated to speak with one voice, representatives from the International Confederation of Midwives (ICM), European Midwives’ Association (EMA) and European Forum for National Nursing and Midwifery Associations (EFNNMA), organizations which bring together all the countries of the EU/EEA, prepared templated responses for national stakeholders to share with the tenderer in November. In these responses we express our concerns about the process and provide a way forward for the update to the Directive based on Global Standards. According to ICM’s records, at least 96 organizations used this template in their stakeholder response to the tenderer (55 professional associations, 33 educational institutions, 8 regulators from a total of 24 countries).

This collaboration critically shows the importance of the Directive update to the development of the midwifery profession in Europe, and beyond. The final version of our joint suggested changes to the Directive can be found in the Supplementary file.

## The EU Directive is critical

The EU Directive on Midwifery is designed to set the minimum standard for midwifery education and scope of practice across all EU/EEA countries. However, it often functions as a ceiling.

It is critical that updates to the Directive are harmonized with the ICM Essential Competencies for Midwifery Practice, and Global Standards for Regulation and Education – the global standards for the profession. These standards are regularly updated and endorsed by ICM’s Council, made up of midwives’ associations from 131 countries, which, at the time of writing, represent 29 of 30 EU/EEA countries. Neglecting to align the Directive to Global Standards weakens efforts to harmonize and elevate midwifery practice across Europe, creating disparities and limiting the potential for mutual recognition and mobility. Finally, the Directive has implications even outside EU/EEA countries – many countries look to the EU Directive as a benchmark when developing or updating their midwifery standards for scope of practice, education and regulation.

## Implications beyond professional standards

Research shows that midwives educated to the ICM Global Standards for Education can provide care for over 90% of women’s sexual, reproductive, maternal, newborn and adolescent health (SRMNAH) needs^14^. When they are enabled, midwives transform health outcomes, with lower mortality and morbidity rates among mothers and newborns, reduce over medicalization, and ensure more positive experiences, all while being more cost-effective and less resource-intensive for health systems^15^.

For example, not having a robust, evidence-based Directive means that in many EU/EEA countries, midwives still lack legal medication prescribing rights, even though evidence shows midwives’ prescribing is safe, effective, and in line with international recommendations and the ICM Essential Competencies^16-18^. Not having prescribing rights means that midwives cannot practice their profession independently and cannot provide comprehensive care to women and newborns. This is but one example of how health systems in EU/EEA countries are not utilizing midwives to the extent that they can and should.

## Intersectionality

The profession of midwifery is affected by intersectional discrimination; on the one hand, gender equality is a mostly female profession that cares for women and vulnerable newborns, on the other hand, midwifery care is a cost-saving and health outcome-optimizing intervention that often operates within health systems where competing professional interests, including those from obstetricians, especially in private practice or working in private facilities, can influence policy and regulatory environments in ways that limit the extent to which midwives are enabled to practise independently.

Finally, it is important to note that the countries that go beyond the minimum standards set by the EU Directive are those with a higher Gender Equality Index (GEI), while those who use the Directive as the maximum standard have a lower GEI score^19^.

Despite the proven benefits midwives provide^20^, the enabling environment midwives need is often pushed back upon by countries and health systems. This is especially true during periods when women’s health and rights become politicized or when health authorities are looking for short-term financial savings without considering the strategic, long-term importance of a strong and autonomous midwifery workforce. Modernizing the EU Directive on Midwifery would help ensure that midwives all over the EU/EEA can provide the critical services we all need, leveraging modern tools and practices in line with the EU’s broader digital transformation in healthcare and system resilience strategies. Furthermore, updating the Directive in line with scientific knowledge and international standards is now a part of the EU Gender Equality Strategy^21^.

We also note that the methodology and process used in updating the Directive reflects these intersecting forms of discrimination rather than taking a gender transformative approach based on scientific evidence.

## The future of health in Europe

Investing in quality midwifery education in Europe is an investment in the future, both in terms of health and economic development. It empowers more women to participate in the workforce and increases access to respectful, high-quality SRMNAH services. However, health systems often deprioritize these investments – particularly during periods of political or fiscal instability. Modernizing the Directive ensures resilience, equity, and preparedness in alignment with EU strategies and international standards.

Countries with strong, well-resourced midwifery education programs that meet the ICM Global Standards have historically had higher birth rates, better SRMNAH outcomes, and better access for more women and families to SRMNAH services^22^. A strong midwifery profession empowers women, uplifts communities, and contributes to broader economic growth by enabling women to have access to SRMNAH services, and positive childbirth experiences, participate in the workforce and ensure quality, respectful care and healthy families. Midwives are also pivotal in upholding reproductive agency, critical in a region where the choice to freely decide how many children women will have is made difficult – either because women do not have access to abortion services^23^, or because social policies make it so they cannot have the (higher) number of children they want^20^. In a world where one in six people faces infertility, midwives’ role in supporting sexual and reproductive health is critical to prevent some types of infertility or subfertility^20^, contributing to sustainable demographic policies within the EU/EEA.

Furthermore, in the digital landscape, midwives’ role extends to navigating a complex environment where misinformation and information gaps pose significant challenges for both professionals and the families they serve^24,25^. The prevalence of unreliable digital narratives can severely erode women’s trust in the established health system and professional advice. Consequently, midwives are instrumental not only in identifying credible resources and actively dispelling untruths but, more critically, in rebuilding trust by providing accurate information and fostering confidence in evidence-based midwifery care^26-28^.

It is therefore critical that updates to the EU Directive on Midwifery transcend mere bureaucratic processes; they must reflect the extensive body of midwifery evidence, incorporate scientific and technological advancements, and align with international standards set by bodies like ICM and other global authorities, like WHO and UNFPA^7,29,30^. They must also reflect the EU’s commitment to patient safety and gender equality. Ultimately, updating the EU Directive on Professional Qualifications: Midwife, to robustly reflect contemporary scientific evidence and global standards as the optimal path forward.

## Conclusion

Each of us shares the experience of being born, and the care from our mothers shapes the trajectory of our life’s course. If we value having resilient, healthy societies, we should value the midwives that make it possible for women and newborns to have the services they need, no matter where they live in the region.

The EU Directive on Minimum Professional Standards: Midwife affects every life in the European Union, and, as such, impacts the strength of the Union. Having a strong Directive to lead the profession is critical for national alignment, freedom of movement, and healthy futures.

## Data Availability

Data sharing is not applicable to this article as no new data were created.

## References

[1] Directive 2005/36/EC of the European Parliament and of the Council of 7 September 2005 on the recognition of professional qualifications. Official Journal of the European Union. 2005;L255:22-142. Accessed April 8, 2026. https://eur-lex.europa.eu/eli/dir/2005/36/oj/eng

[2] Special report 10/2024: The recognition of professional qualifications in the EU – An essential mechanism, but used sparsely and inconsistently. European Court of Auditors. Accessed April 8, 2026. https://www.eca.europa.eu/en/publications/SR-2024-10&od=1

[3] Vermeulen J, Luyben A, Jokinen M, Matintupa E, O’Connell R, Bick D. Establishing a Europe-wide foundation for high quality midwifery education: the role of the European Midwives Association (EMA). Midwifery. 2018;64:128-131. doi:10.1016/j.midw.2018.06.009 29970310

[4] European Parliament. Report on the situation of sexual and reproductive health and rights in the EU, in the frame of women’s health (A9-0169/2021). Updated June 2021. Accessed April 8, 2026. https://www.europarl.europa.eu/doceo/document/A-9-2021-0169_EN.html

[5] European Commission: Directorate-General for Justice and Consumers, Scientific Analysis and Advice on Gender Equality in the EU (SAAGE), Fondazione Giacomo Brodolini (FGB). Obstetric violence in the European Union: Situational analysis and policy recommendations. Publications Office of the European Union; 2024. Accessed April 8, 2026. https://data.europa.eu/doi/10.2838/440301

[6] European Commission. The EU Roadmap for Women’s Rights: a renewed push for gender equality. March 7, 2025. Accessed April 8, 2026. https://commission.europa.eu/news-and-media/news/eu-roadmap-womens-rights-renewed-push-gender-equality-2025-03-07_en

[7] International Confederation of Midwives. Essential Competencies for Midwifery Practice. Updated September 19, 2024. Accessed April 8, 2026. https://internationalmidwives.org/resources/essential-competencies-for-midwifery-practice/

[8] International Confederation of Midwives. Global Standards for Midwifery Education. Updated January 4, 2021. Accessed April 8, 2026. https://internationalmidwives.org/resources/global-standards-for-midwifery-education/10.1016/j.midw.2011.04.001 21550149

[9] World Health Organization. Implementation guidance on transitioning to midwifery models of care. June 17, 2025. Accessed April 8, 2026. https://www.who.int/publications/i/item/9789240110199

[10] European Commission. EU Funding & Tenders Portal. Mapping and Assessment of Developments for One of Sectoral Professions under Directive 2005/36/EC – Midwife. Accessed April 8, 2026. https://ec.europa.eu/info/funding-tenders/opportunities/portal/screen/opportunities/tender-details/14894

[11] Spark Legal and Policy Consulting. Discussion Paper for the Workshop: EU minimum harmonised training for midwives – time for an update? Spark Legal and Policy Consulting; 2024. Accessed April 8, 2026. https://www.sparklegalpolicy.eu/wp-content/uploads/2024/10/Workshop-Midwives-Discussion-paper.pdf

[12] European Commission: Directorate-General for Employment, Social Affairs and Inclusion, Spark Legal and Policy Consulting. Mapping and assessment of developments of one of the sectoral professions under Directive 2005/36/EC: The profession of midwife. Publications Office of the European Union; 2025. Accessed April 8, 2026. https://data.europa.eu/doi/10.2767/1436858

[13] European Commission. Commission work programme 2026. October 21, 2025. Accessed April 8, 2026. https://commission.europa.eu/strategy-and-policy/strategy-documents/commission-work-programme/commission-work-programme-2026_en

[14] United Nations Population Fund. The State of the World’s Midwifery 2021. United Nations Population Fund. Accessed April 8, 2026. https://www.unfpa.org/sowmy

[15] The Lancet. Midwifery. Accessed April 8, 2026. https://www.thelancet.com/series-do/midwifery

[16] World Health Organization. Medication Without Harm; 2017. Accessed April 8, 2026. https://iris.who.int/bitstream/handle/10665/255263/WHO-HIS-SDS-2017.6-eng.pdf

[17] European Union. Directive 2012/26/EU of the European Parliament and of the Council of 25 October 2012 amending Directive 2001/83/EC as regards pharmacovigilance. Official Journal of the European Union. October, 27, 2012. Accessed April 8, 2026. https://eur-lex.europa.eu/eli/dir/2012/26/oj/eng

[18] Conseil National de l’Ordre des Sages-Femmes. Bienvenue sur le site de l’Ordre des sages-femmes. Accessed April 8, 2026. https://www.ordre-sages-femmes.fr

[19] European Institute for Gender Equality. Gender Equality Index. Accessed April 8, 2026. https://eige.europa.eu/gender-equality-index/2024

[20] United Nations Population Fund. The real fertility crisis: The pursuit of reproductive agency in a changing world. State of World Population; 2025. Accessed April 8, 2026. https://www.unfpa.org/swp2025

[21] European Parliament. European Parliament resolution of 13 November 2025 on the Gender Equality Strategy 2025 (2024/2125(INI)). November 13, 2025. Accessed April 8, 2026. https://www.europarl.europa.eu/doceo/document/TA-10-2025-0278_EN.html

[22] Clark EV, LaNoue M, Clouse K, Zuber A, Neal J. Development of a midwifery regulatory environment index using data from the Global Midwives’ Associations map survey. BMC Health Serv Res. 2025;25(1):728. doi:10.1186/s12913-025-12694-w 40394621 10.1186/s12913-025-12694-w PMC12093735

[23] European Parliament. ‘My Voice, My Choice’: MEPs support citizens’ initiative on accessible abortion. December 17, 2025. Accessed April 8, 2026. https://www.europarl.europa.eu/news/en/press-room/20251211IPR32167/my-voice-my-choice-meps-support-citizens-initiative-on-accessible-abortion

[24] European Commission. (n.d.). eHealth - digital services in health and care. Accessed April 8, 2026. https://digital-strategy.ec.europa.eu/en/policies/ehealth

[25] European Commission. Digital health and care. Accessed April 8, 2026. https://health.ec.europa.eu/ehealth-digital-health-and-care/digital-health-and-care_en

[26] European Commission. Managing health data. Accessed April 8, 2026. https://digital-strategy.ec.europa.eu/en/policies/health-data

[27] DigitalHealthEurope. DigitalHealthEurope recommendations on the European Health Data Space; 2021. Accessed April 8, 2026. https://digitalhealtheurope.eu/wp-content/uploads/DHE_recommendations_on_EHDS_July_2021.pdf

[28] DIGITALEUROPE. DIGITALEUROPE Executive Council for Health’s recommendations for EU digital health policy (2024-29). Accessed April 8, 2026. https://cdn.digitaleurope.org/uploads/2024/02/DIGITALEUROPE-recommendations-EU-digital-health-policy-2024-29-policy-paper.pdf

[29] World Health Organization. Transitioning to midwifery models of care: global position paper. October 15, 2024. Accessed April 8, 2026. https://www.who.int/publications/i/item/9789240098268

[30] United Nations Population Fund. UNFPA and Partners Launch Midwifery Accelerator to Confront Maternal Health Crisis. April 7, 2025. Accessed April 8, 2026. https://www.unfpa.org/press/unfpa-and-partners-launch-midwifery-accelerator-confront-maternal-health-crisis

